# Three-dimensional spatial localization and volume estimation of prostate tumors using ^18^F-PSMA-1007 PET/CT versus multiparametric MRI

**DOI:** 10.1007/s00259-024-07021-0

**Published:** 2024-12-27

**Authors:** Guocheng Huang, Patrick Albers, Nikhile Mookerji, Tyler Pfanner, Amaris Hui, Rohan Mittal, Stacey Broomfield, Lucas Dean, Blair St. Martin, Niels-Erik Jacobsen, Howard Evans, Yuan Gao, Ryan Hung, Jonathan Abele, Peter Dromparis, Joema Felipe Lima, Tarek A. Bismar, Evangelos Michelakis, Gopinath Sutendra, Frank Wuest, Wendy Tu, Benjamin A. Adam, Christopher Fung, Sunita Ghosh, Alexander Tamm, Adam Kinnaird, The Next Generation Trial Investigators, Guocheng Huang, Patrick Albers, Nikhile Mookerji, Tyler Pfanner, Amaris Hui, Rohan Mittal, Stacey Broomfield, Lucas Dean, Blair St. Martin, Niels-Erik Jacobsen, Howard Evans, Yuan Gao, Ryan Hung, Jonathan Abele, Peter Dromparis, Joema Felipe Lima, Tarek A. Bismar, Evangelos Michelakis, Gopinath Sutendra, Frank Wuest, Wendy Tu, Benjamin A. Adam, Christopher Fung, Sunita Ghosh, Alexander Tamm, Adam Kinnaird

**Affiliations:** 1 Division of Urology, Department of Surgery, University of Alberta, Edmonton, Canada; 2 Department of Radiology & Diagnostic Imaging, University of Alberta, Edmonton, Canada; 3 Department of Laboratory Medicine & Pathology, University of Alberta, Edmonton, Canada; 4 Alberta Centre for Urologic Research and Excellence (ACURE), Edmonton, Canada; 5 Departments of Pathology & Laboratory Medicine, Oncology, Biochemistry and Molecular Biology, University of Calgary, Cumming School of Medicine, Calgary, Canada; 6 Alberta Prostate Cancer Research Initiative (APCaRI), Edmonton, Canada; 7 Department of Medicine, University of Alberta, Edmonton, Canada; 8 Department of Oncology, University of Alberta, Edmonton, Canada; 9 Cancer Research Institute of Northern Alberta (CRINA), Edmonton, Canada

**Keywords:** Diagnostic imaging, Prostatic neoplasms, PET-CT, Multiparametric MRI, Tumor volume

## Abstract

**Purpose:**

Fluorine-18 prostate-specific membrane antigen-1007 positron emission tomography/computed tomography (^18^F-PSMA-1007 PET/CT) has been shown to be superior to multiparametric magnetic resonance imaging (MRI) for the locoregional staging of intermediate-risk and high-risk prostate tumors. This study aims to evaluate whether it is also superior in estimating tumor parameters, such as three-dimensional spatial localization and volume.

**Methods:**

134 participants underwent ^18^F-PSMA-1007 PET/CT and MRI prior to radical prostatectomy as part of the validating paired-cohort Next Generation Trial (NCT05141760). MRI, ^18^F-PSMA-1007 PET/CT, and final pathology were independently assessed by blinded radiologists, nuclear medicine physicians, and pathologists, respectively. Individual tumor nodules were measured in three dimensions and cognitively registered to 38 segment prostate diagrams as per PI-RADSv2.1. Correct spatial localization was compared using McNemar test and estimation of tumor volumes were compared using linear regression and partial F-test.

**Results:**

286 tumor nodules were identified by final histopathology. ^18^F-PSMA-1007 PET/CT was superior to MRI for correct localization (186 [65.0%] vs 134 [46.9%], p < 0.001) and tumor volume estimation (R^2^ = 0.545 vs 0.431, p < 0.001). Larger tumors and higher Gleason Grade Group (GGG) were associated with correct localization by ^18^F-PSMA-1007 PET/CT (OR = 2.05, p < 0.001 for tumor volume and OR = 4.92, p < 0.01 for ≥ GGG3) and MRI (OR = 1.81, p < 0.001 for tumor volume and OR = 11.67, p < 0.001 for ≥ GGG3).

**Conclusion:**

^18^F-PSMA-1007 PET/CT outperforms MRI for determination of three-dimensional spatial localization and volume of prostate tumors. These findings support the use of ^18^F-PSMA-1007 PET/CT prior to definitive treatment of localized prostate cancers.

**Supplementary Information:**

The online version contains supplementary material available at 10.1007/s00259-024-07021-0.

## Introduction

Multiparametric magnetic resonance imaging (MRI) has been the gold standard imaging modality for the primary staging of localized prostate cancer for the last decade [1, 2]. We conducted the Next Generation Trial, which was a validating paired-cohort trial comparing fluorine-18 prostate-specific membrane antigen-1007 PET/computed tomography (^18^F-PSMA-1007 PET/CT) to MRI for the primary locoregional staging of intermediate and high-risk prostate cancers in men undergoing radical prostatectomy [3]. We showed that ^18^F-PSMA-1007 PET/CT was superior to MRI for the primary tumor (‘T’) staging of prostate cancer. However, differences in the intraprostatic spatial localization of tumors and accuracy of tumor volume measurements between imaging techniques remains unknown.

Accurate estimation of tumor volume and localization has important implications for treatment planning such as focal therapy margins, nerve-sparing during prostatectomy, and radiation boosting to the dominant nodule (i.e. the ‘Index’ lesion) [4, 5]. MRI has been shown to underestimate tumor volume by 300% and identifies fewer non-dominant tumors in multifocal disease [3, 6]. ^18^F-PSMA-1007 PET/CT has enhanced spatial resolution and minimal urinary excretion compared to other PSMA radioligands [7]. Therefore, we hypothesized that ^18^F-PSMA-1007 PET/CT may be superior to MRI for tumor characteristics such as intraprostatic localization and volume estimation. To explore this, we performed a secondary analysis of data from the Next Generation Trial, using final pathology after radical prostatectomy as the ground truth.

## Materials and methods

### Participants and trial design

As previously published, 134 participants underwent ^18^F-PSMA-1007 PET/CT, and multiparametric MRI scans (detailed imaging protocols available in Supplementary Methods) within two weeks of one another, followed by robot-assisted laparoscopic radical prostatectomy between March 2021 and June 2023 (NCT05141760) [3]. The trial received regional Human Research Ethics approval under protocol HREBA.CC-21–0073 and was monitored by the Northern Alberta Clinical Trials and Research Centre. Radiologists, nuclear medicine physicians, and pathologists interpreting the data were blinded to other clinical, imaging, and pathology data. MRI sequences were interpreted using PI-RADS v2.1 criteria [8], while ^18^F-PSMA-1007 PET/CT sequences were interpreted using modified PROMISE guidelines [3, 9]. MRI interpretations were performed by consensus reading by at least two radiologists (fellowship trained body radiologists had each performed at least 1500 previous prostate MRI interpretations) and ^18^F-PSMA-1007 PET/CT interpretations were performed by consensus reading by a Nuclear Medicine physician and senior combined radiology and nuclear medicine resident (fewer than 100 previous ^18^F-PSMA-1007 PET/CT interpretations collectively). Final histopathology was centrally reviewed. The trial design was a validating paired-cohort trial (Oxford level of evidence 1b for diagnostic trials).

## Nodule-based segmental and tumor volume analyses

As prostate cancer is a multifocal disease, each tumor nodule in the prostate was independently assessed by the radiologists, nuclear medicine physicians, and pathologists. We included the dominant nodule (determined by Gleason Grade Group (GGG) and size) and up to 3 non-dominant nodules in our analysis per patient. We used the 38-segment prostate schematic as per PI-RADS v2.1 (41 segment when including seminal vesicles and external urethral sphincter) to determine tumor nodule localization. In summary, each prostate is divided into left/right and medial (m) and lateral (l) on axial sections; into the apex, mid-gland, and base in the transverse plane; and anterior (a) and posterior (p) in the coronal plane. Considering the prostate zones (TZ, CZ, PZ, and AFS), the base in each hemigland can be further divided into AFS, TZa, TZp, PZa, PZpm, PZpl, and CZ, while both the mid-gland and apex can be divided into AFS, TZa, TZp, PZa, PZpm, and PZpl, respectively [8].

Nodules detected by ^18^F-PSMA-1007 PET/CT and MRI were compared to pathology, which was considered the ground truth (Supplementary Fig. 1). To be considered accurately detected, nodules observed on imaging had to occupy at least one segment that overlapped with pathology nodules in any direction (Supplementary Fig. 2). Tumor nodule volume was calculated based on PI-RADS v2.1 criteria using maximum measurements in anterior–posterior (AP), transverse (TV), and craniocaudal (CC) planes on axial (AP/TV) and coronal (CC), via the previously validated formula maximum AP x TV x CC × 0.52 [8]. Tumor nodule was assessed by multiparametric MRI and the maximum tumor extents were measured on T2-weighted images. On ^18^F-PSMA-1007 PET/CT, it was defined using a semi-automated Oasis software (Segami Corporation) measurement tool which identified tissue with greater than 30% maximum standardized uptake value for grade 2 or 3 nodules and 50% to 65% maximum standardized uptake value for grade 1 nodules within a selected sphere [3]. Initial measurements were confirmed by a second review. Nodule detection rates in both imaging methods were stratified further by nodule volume, dominant vs. non-dominant, Gleason Grade Group, focality, and three-dimensional localization.

## Statistical analysis

The comparison of nodule detection between ^18^F-PSMA-1007 PET/CT and MRI was analyzed by the McNemar test. The dominant nodule volume and non-dominant tumor volumes were analyzed. Linear regression analysis was used to estimate volume prediction abilities of two imaging modalities. The partial F-test was used for the comparison of the two linear regression lines. Univariate and multivariate analysis were used for the identification of variables that influence the nodule detection rates in either imaging test. Two-sided p value of 0.05 or less indicated statistical significance. Analyses were performed using STATA software, version 18 (College Station, Texas).

## Results

### Spatial localization of tumor nodules

**Fig. 1:**
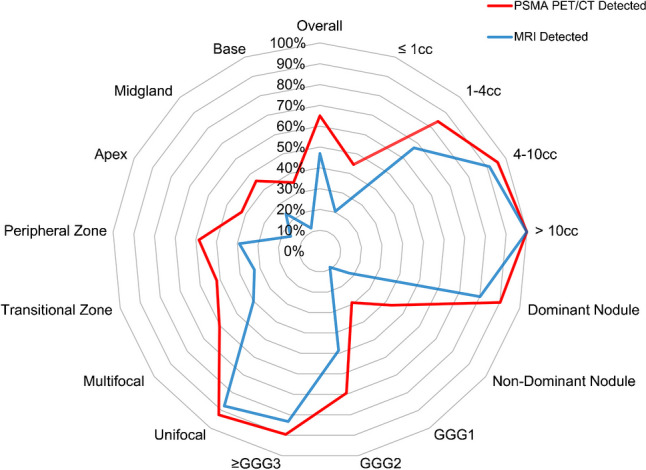
Radar plot of correct spatial localization by ^18^F-PSMA-1007 PET/CT and MRI stratified by tumor size, dominant vs. non-dominant nodules, GGG (Gleason Grade Group), focality, and prostate zones

In total, 286 tumor nodules were identified by final histopathology. ^18^F-PSMA-1007 PET/CT outperformed MRI in correctly identifying prostate tumor nodule locations (186 [65.0%] vs 134 [46.9%], p < 0.001, Table 1). Stratified analysis showed higher tumor nodule detection by ^18^F-PSMA-1007 PET/CT compared to MRI for tumor volumes ≤ 1 cc (70 [44.6%] vs 32 [20.4%], p < 0.001) and 1 to 4 cc (59 [84.3%] vs 47 [67.1%], p < 0.01), but no significant difference for larger volumes (4–10 cc or larger). ^18^F-PSMA-1007 PET/CT exhibited better detection than MRI for both dominant nodules (120 [90.2%] vs 107 [80.5%], p = 0.01) and non-dominant nodules (66 [43.1%] vs 27 [17.6%], p < 0.001) in segmental analysis. ^18^F-PSMA-1007 PET/CT detected more GGG 1 nodules than MRI (16 [29.1%] vs 5 [9.1%], p = 0.01) and GGG 2 (127 [69.4%] vs 89 [48.6%], p < 0.001) nodules, while showing similar detection rates for GGG 3 or higher nodules (43 [89.6%] vs 40 [83.3%], p = 0.45). Additionally, ^18^F-PSMA-1007 PET/CT detected more multifocal nodules compared to MRI (149 [60.6%] vs 99 [40.2%], p < 0.001). Across different dimensions and zones, including transition, peripheral, apex, mid-gland, anterior, posterior, medial, and lateral locations, ^18^F-PSMA-1007 PET/CT demonstrated superior detection over MRI (Table 1). In summary, ^18^F-PSMA-1007 PET/CT was at least as good as or superior to MRI across all stratifications tested (Fig. 1).

**Table 1:** Correct identification of three-dimensional localization of tumor nodules (n = 286) by ^18^F-PSMA-1007 PET/CT and MRI

	^18^F-PSMA-1007 PET/CT		MRI		p-values
	Detected (n = 186)	Missed (n = 100)	Detected (n = 134)	Missed (n = 152)	p < 0.001
Tumor Volume(cc)					
≤ 1 (n = 157)	70 (44.6)	87 (55.4)	32 (20.4)	125 (79.6)	** < 0.001**
1–4 (n = 70)	59 (84.3)	11 (15.7)	47 (67.1)	23 (32.9)	** < 0.01**
4–10 (n = 45)	43 (95.6)	2 (4.4)	41 (91.1)	4 (8.9)	0.69
> 10 (n = 14)	14 (100)	0 (0)	14 (100)	0 (0)	1
Nodule Status					
Dominant (n = 133)	120 (90.2)	13 (9.8)	107 (80.5)	26 (19.5)	**0.01**
Non-Dominant (n = 153)	66 (43.1)	87 (56.9)	27 (17.6)	126 (82.4)	** < 0.001**
Gleason Grade Group					
1 (n = 55)	16 (29.1)	39 (70.9)	5 (9.1)	50 (90.9)	**0.01**
2 (n = 183)	127 (69.4)	56 (30.6)	89 (48.6)	94 (51.4)	** < 0.001**
≥ 3 (n = 48)	43 (89.6)	5 (10.4)	40 (83.3)	8 (16.7)	0.45
Tumor focality					
Unifocal (n = 40)	37 (92.5)	3 (7.5)	35 (87.5)	5 (12.5)	0.68
Multifocal (n = 246)	149 (60.6)	97 (39.4)	99 (40.2)	147 (59.8)	** < 0.001**
Zone Involved					
Transition (n = 58)	30 (51.7)	28 (48.3)	19 (32.8)	39 (67.2)	**0.02**
Peripheral (n = 159)	93 (58.5)	66 (41.5)	62 (39.0)	97 (61.0)	** < 0.001**
Both (n = 69)	63 (91.3)	6 (8.7)	53 (76.8)	16 (23.2)	** < 0.01**
Transverse Plane					
Apex (n = 38)	16 (42.1)	22 (57.9)	6 (15.8)	32 (84.2)	**0.01**
Midgland (n = 90)	41 (45.6)	49 (54.4)	22 (24.4)	68 (75.6)	** < 0.001**
Base (n = 17)	6 (35.3)	11 (64.7)	2 (11.8)	15 (88.2)	0.13
≥ 2 Areas (n = 141)	123 (87.2)	18 (12.8)	104 (73.8)	37 (26.2)	** < 0.001**
Coronal Plane					
Anterior (n = 85)	43 (50.6)	42 (49.4)	25 (29.4)	60 (70.6)	** < 0.001**
Posterior (n = 124)	76 (61.3)	48 (38.7)	56 (45.2)	68 (54.8)	** < 0.001**
Cross A-P Midline (n = 77)	67 (87.0)	10 (13.0)	53 (68.8)	24 (31.2)	** < 0.01**
Sagittal Plane					
Medial (n = 75)	37 (49.3)	38 (50.7)	25 (33.3)	50 (66.7)	**0.02**
Lateral (n = 94)	53 (56.4)	41 (43.6)	26 (27.7)	68 (72.3)	** < 0.001**
Cross M-L Midline (n = 117)	96 (82.1)	21 (17.9)	83 (70.9)	34 (29.1)	**0.02**

Data are presented as n (%)

## Tumor volume estimation

**Fig. 2:**
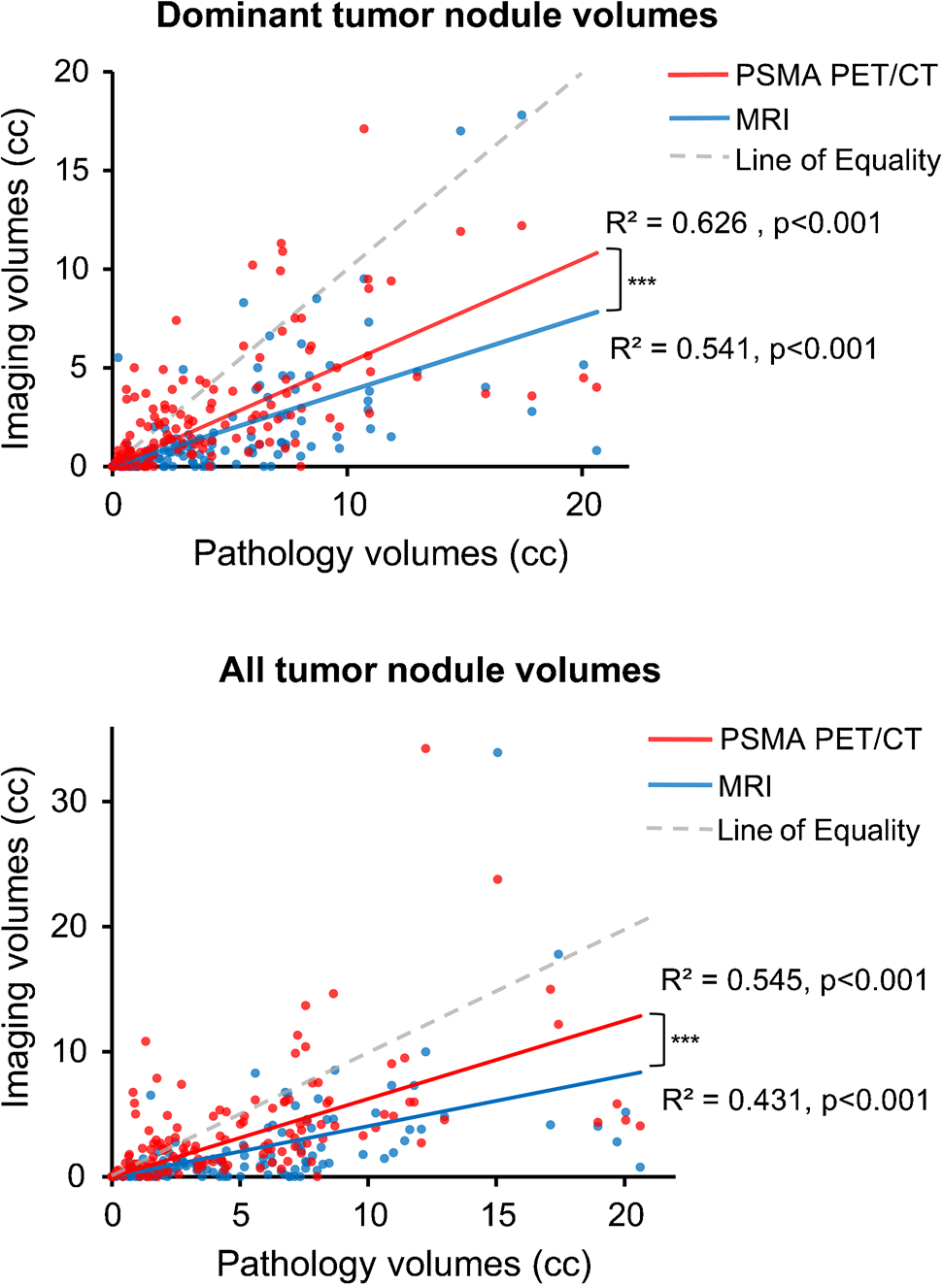
Scatter plot and linear regression of estimated tumor volumes by ^18^F-PSMA-1007 PET/CT (red) and MRI (blue). Dominant tumor nodule estimation on top and all tumor nodules estimation on bottom. ***p < 0.001, ^18^F-PSMA-1007 PET/CT superior to MRI for estimation of tumor volumes

MRI predictions of dominant tumor nodule volumes (R^2^ = 0.541, p < 0.001) and all tumor nodule volumes (R^2^ = 0.431, p < 0.001) were positively correlated with pathological volumes (Fig. 2). Similarly, ^18^F-PSMA-1007 PET/CT predictions of dominant nodule volume (R^2^ = 0.626, p < 0.001) and all tumor nodule volumes (R^2^ = 0.545, p < 0.001) were positively correlated with pathological volumes. Comparing imaging techniques, ^18^F-PSMA-1007 PET/CT was more accurate than MRI in predicting dominant nodule volumes (p < 0.001) and all tumor nodule volumes (p < 0.001). ^18^F-PSMA-1007 PET/CT predictions were more likely to fall within 50%−150% of both dominant nodule volume (42.1% vs 21.8%, p < 0.001) and all tumor nodule volumes (41.6% vs 19.8%, p < 0.001) compared to MRI (Table 2).

**Table 2:** Relative estimation of tumor volume by ^18^F-PSMA-1007 PET/CT and MRI compared to pathological volume

Relative Tumor Volumes (Dominant Nodule)		MRI		
		50%−150%	< 50% or > 150%	p-value
^18^F-PSMA-1007 PET/CT	50%−150%	15 (11.3)	41 (30.8)	** < 0.001**
	< 50% or > 150%	14 (10.5)	63 (47.4)	
Relative Tumor Volumes (All Nodules)		MRI		
		50%−150%	< 50% or > 150%	p-value
^18^F-PSMA-1007 PET/CT	50%−150%	16 (12.3)	39 (29.3)	** < 0.001**
	< 50% or > 150%	10 (7.5)	68 (51.1)	

Data are presented as n (%)

## Multivariable analysis of prostate tumor detection by ^18^F-PSMA-1007 PET/CT and MRI

On multivariate analysis for tumor detection, larger tumors had a higher likelihood of being identified by ^18^F-PSMA-1007 PET/CT (OR = 2.05, p < 0.001) and MRI (OR = 1.81, p < 0.001) (Table 3). Similarly, higher GGG increased the likelihood of detecting tumors with both ^18^F-PSMA-1007 PET/CT (for ≥ GGG3 OR = 4.92, p < 0.01) and MRI (for ≥ GGG3 OR = 11.67, p < 0.001). The presence of bilateral tumor nodules significantly reduced correct tumor identification on both sides of the gland by MRI (OR = 0.15, p = 0.02) but not ^18^F-PSMA-1007 PET/CT (OR = 0.25, p = 0.11).

**Table 3:** Multivariable analysis for correct tumor localization by ^18^F-PSMA-1007 PET/CT and MRI

	^18^F-PSMA-1007 PET/CT		MRI	
	OR	p-value	OR	p-value
Tumor Volume	**2.05**	** < 0.001**	**1.81**	** < 0.001**
T stage		0.60		0.33
≥ pT3	1.17		1.38	
pT2	1.00		1.00	
Tumor Focality		0.88		0.33
Multifocal	0.89		0.54	
Unifocal	1.00		1.00	
Tumor Laterality		0.11		**0.02**
Bilateral	0.25		**0.15**	
Unilateral	1.00		**1.00**	
Gleason Grade Group		** < 0.01**		** < 0.001**
≥ 3	**4.92**		**11.67**	
2	**2.27**		**3.19**	
1	**1.00**		**1.00**	

OR = odds ratio. See Supplementary Table 1 for univariate analysis for age, pre-operative PSA, prostate volume, tumor volume, T stage, tumor focality, and Gleason Grade Group. Age, pre-operative PSA, prostate volume, and tumor volume were analyzed as continuous variables for each increasing increment (year, ng/ml, cc)

## Discussion

We found that ^18^F-PSMA-1007 PET/CT was superior to MRI for the intraprostatic spatial localization of prostate tumors and for the estimation of tumor volumes. This superiority of ^18^F-PSMA-1007 PET/CT was true for localization and volume estimation of both dominant nodules and non-dominant nodules. Increased pathological tumor size and higher Gleason Grade Group increased the odds of correctly identifying the location of the tumor for both imaging modalities.

Although MRI tumor volume was positively correlated with pathology tumor volume, it has been previously shown to be a poor estimator [6, 10, 11]. In this trial, MRI correctly estimated (within 50% of pathological volumes) tumor size in only 19.8% of nodules. In comparison, ^18^F-PSMA-1007 PET/CT correctly estimated tumor size in 41.6% of nodules. These results suggest that ^18^F-PSMA-1007 PET/CT is better than MRI for measuring intraprostatic tumor burden. This has important implications for tumor treatment margin determination during focal therapy procedures as most margins are currently based on MRI features.

There is evidence that expression of PSMA increases with increasing Gleason Grade Group [12]. Similarly, sensitivity of multiparametric MRI for prostate cancer detection increases with higher Gleason Grade Group [13]. In this trial, increased Gleason Grade Group was independently associated with the correct localization of tumor nodules. Grade Group 3 or higher tumors were accurately detected in 90% and 83% of cases using ^18^F-PSMA-1007 PET/CT and MRI, respectively. However, these numbers significantly decreased for Gleason Grade Group 2 tumors, being detected in only 69% and 49% of cases by ^18^F-PSMA-1007 PET/CT and MRI, respectively. This means that MRI fails to correctly determine tumor location for Gleason Grade Group 2 tumors in over half of cases, which has impact on treatment plans for nerve-sparing, focal therapy and radiation boosting.

Overdiagnosis and overtreatment have been problems with prostate cancer management [14, 15]. An inherent advantage to MRI is reduced detection of Gleason Grade Group 1 tumors [16]. In this trial, ^18^F-PSMA-1007 PET/CT accurately detected Gleason Grade Group 1 tumors in 29% of cases compared to only 9% using MRI. This may lead to an increased rate of false positives for clinically significant prostate cancer when using ^18^F-PSMA-1007 PET/CT compared to MRI.

Both imaging techniques have difficulty in determining when prostate tumors are bilateral or multifocal. ^18^F-PSMA-1007 PET/CT correctly identified 61% of multifocal tumors while MRI identified only 40% of multifocal tumors. This is in keeping with previous results that showed detection of multifocal tumors by MRI in only 39% of cases [17]. Similarly, MRI showed a statistically significant odds ratio of 0.15 for detecting bilaterality of tumor compared to unilaterality. ^18^F-PSMA-1007 PET/CT did not have a statistically significant odds ratio, however it remains low (0.25) for the detection of bilateral tumors. These results emphasize the importance of systematic biopsies, especially on the side contralateral to the dominant nodule, to better understand overall tumor localization, particularly when partial gland treatment is being considered [18–20].

This work has several limitations. These include reliance on cognitive registration using the 38 sector diagrams to determine 3D spatial orientation as well as the utilization of quarter mount pathology for radical prostatectomy specimens. Future studies should explore the genomic landscape of PSMA positive and PSMA negative tumors to determine if the radiomic signatures of PSMA positive tumors portend a worse or better prognosis. Additionally, given the better accuracy of MRI compared to CT for prostate cancer, hybrid PSMA-ligand PET/MRI using a PET/MRI scanner would be an attractive option for future applications.

## Conclusion

In conclusion, in this trial ^18^F-PSMA-1007 PET/CT was superior to MRI for the three-dimensional spatial localization and volume estimation of prostate tumors. These results support the use of ^18^F-PSMA-1007 PET/CT prior to treatment for localized prostate cancers.

## Supplementary Information

Below is the link to the electronic supplementary material.

Supplementary file1 (PPTX 17068 KB)

Supplementary file2 (DOCX 17 KB)

Supplementary file3 (DOCX 19 KB)

## Data Availability

The datasets used and/or analyzed during the current study are available from the corresponding author on reasonable request.
