# Supplementary material for: Three-dimensional spatial localization and volume estimation of prostate tumors using 18F-PSMA-1007 PET/CT versus multiparametric MRI
Source: Eur J Nucl Med Mol Imaging. 2024 Dec 27;52(5):1642–8. doi: 10.1007/s00259-024-07021-0 (PMC11928431; doi:10.1007/s00259-024-07021-0)
Supplement: Supplementary file 1 — Supplementary file1 (PPTX 17068 KB) [file 259_2024_7021_MOESM1_ESM.pptx]

## Slide 1
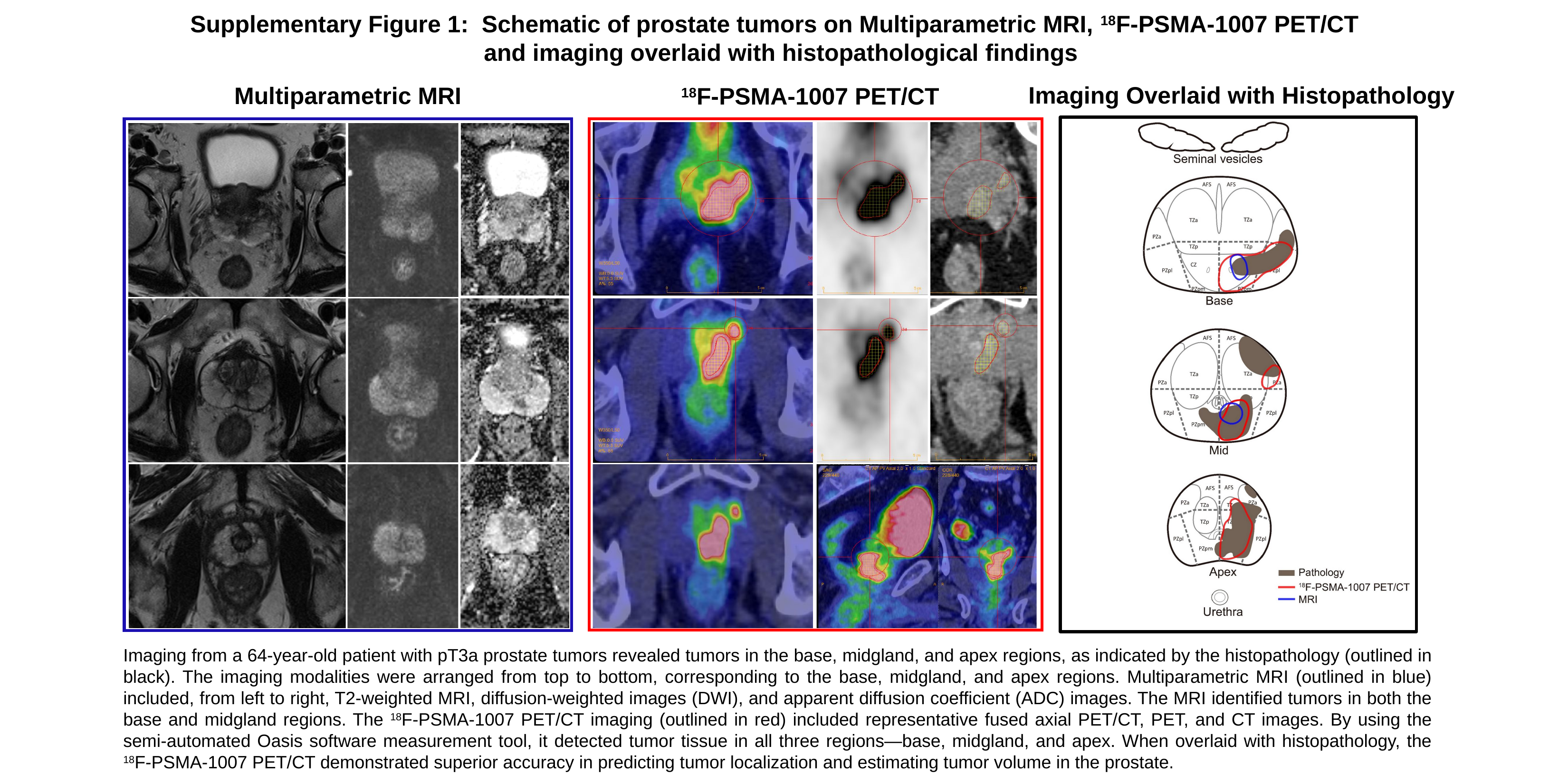

Supplementary Figure 1: Schematic of prostate tumors on Multiparametric MRI, 18F-PSMA-1007 PET/CT
 and imaging overlaid with histopathological findings
 Imaging Overlaid with Histopathology
Multiparametric MRI
18F-PSMA-1007 PET/CT
Imaging from a 64-year-old patient with pT3a prostate tumors revealed tumors in the base, midgland, and apex regions, as indicated by the histopathology (outlined in black). The imaging modalities were arranged from top to bottom, corresponding to the base, midgland, and apex regions. Multiparametric MRI (outlined in blue) included, from left to right, T2-weighted MRI, diffusion-weighted images (DWI), and apparent diffusion coefficient (ADC) images. The MRI identified tumors in both the base and midgland regions. The 18F-PSMA-1007 PET/CT imaging (outlined in red) included representative fused axial PET/CT, PET, and CT images. By using the semi-automated Oasis software measurement tool, it detected tumor tissue in all three regions—base, midgland, and apex. When overlaid with histopathology, the 18F-PSMA-1007 PET/CT demonstrated superior accuracy in predicting tumor localization and estimating tumor volume in the prostate.

## Slide 2
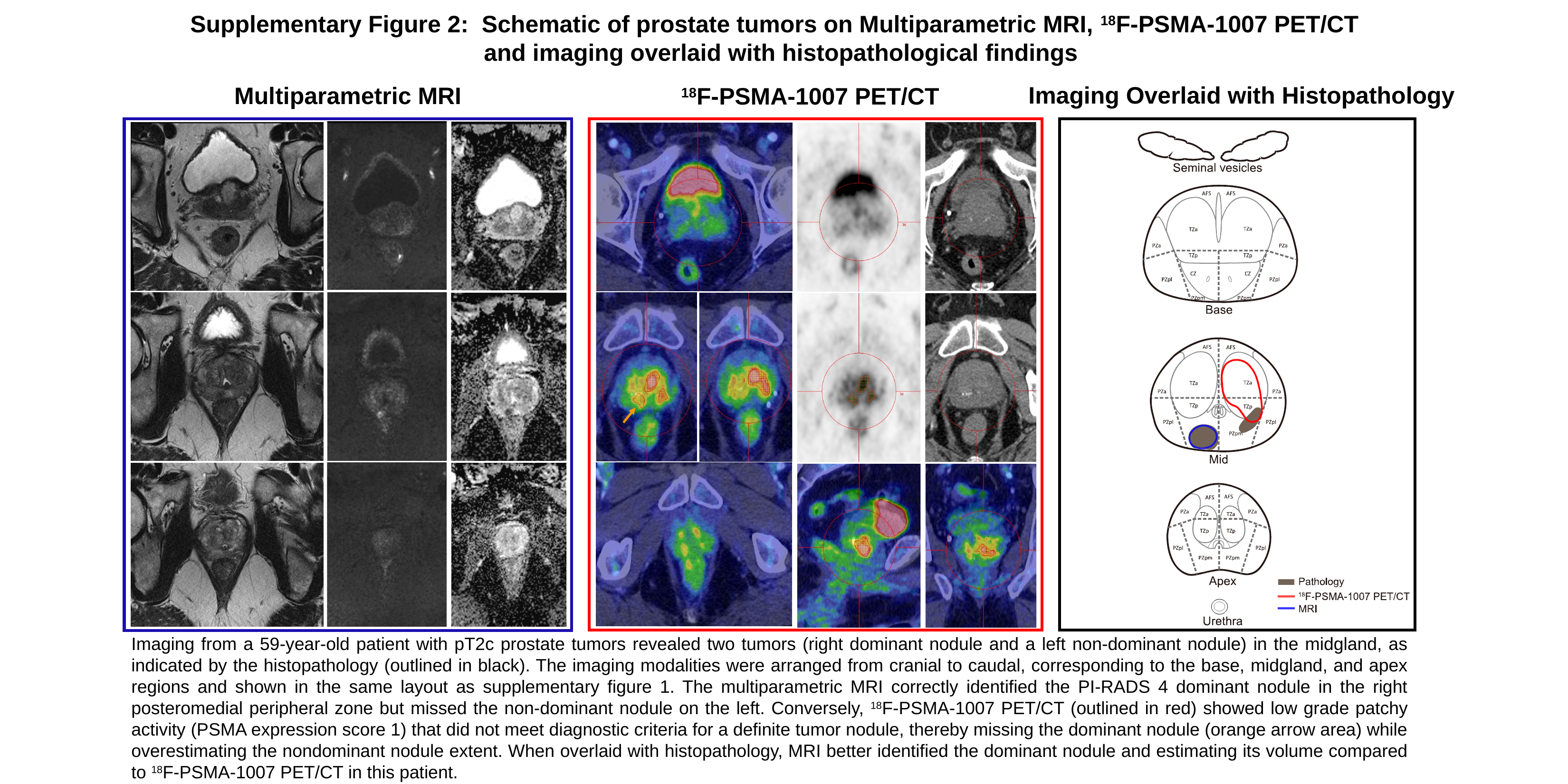

Supplementary Figure 2: Schematic of prostate tumors on Multiparametric MRI, 18F-PSMA-1007 PET/CT
 and imaging overlaid with histopathological findings
 Imaging Overlaid with Histopathology
Multiparametric MRI
18F-PSMA-1007 PET/CT
Imaging from a 59-year-old patient with pT2c prostate tumors revealed two tumors (right dominant nodule and a left non-dominant nodule) in the midgland, as indicated by the histopathology (outlined in black). The imaging modalities were arranged from cranial to caudal, corresponding to the base, midgland, and apex regions and shown in the same layout as supplementary figure 1. The multiparametric MRI correctly identified the PI-RADS 4 dominant nodule in the right posteromedial peripheral zone but missed the non-dominant nodule on the left. Conversely, 18F-PSMA-1007 PET/CT (outlined in red) showed low grade patchy activity (PSMA expression score 1) that did not meet diagnostic criteria for a definite tumor nodule, thereby missing the dominant nodule (orange arrow area) while overestimating the nondominant nodule extent. When overlaid with histopathology, MRI better identified the dominant nodule and estimating its volume compared to 18F-PSMA-1007 PET/CT in this patient.
