# Supplementary material for: Three-dimensional spatial localization and volume estimation of prostate tumors using 18F-PSMA-1007 PET/CT versus multiparametric MRI
Source: Eur J Nucl Med Mol Imaging. 2024 Dec 27;52(5):1642–8. doi: 10.1007/s00259-024-07021-0 (PMC11928431; doi:10.1007/s00259-024-07021-0)
Supplement: Supplementary file 2 — Supplementary file2 (DOCX 17 KB) [file 259_2024_7021_MOESM2_ESM.docx]

The imaging protocols of Multiparametric MRI and ^18^F-PSMA-1007 PET/CT follow the methodology we previously reported in Mookerji N et al., *JAMA Oncology*, 2024.

**Protocol for Multiparametric MRI**

Following manual shim and application of saturation bands across the anterior abdomen, overlying sigmoid colon, and the rectum, magnetic resonance sequences acquired through the pelvis including initial localizer sequences, wide field of view axial T2, high resolution axial/coronal/sagittal T2 of the prostate, axial diffusion-weighted imaging calculated to b1600 with an associated apparent diffusion coefficient map, and axial T1 volumetric interpolated breath-hold examination before and after gadolinium contrast. Postgadolinium T1 volumetric interpolated breath-hold examination sequences were acquired every 11 seconds for a total of 132 seconds. Gadolinium was administered manually over 10 seconds via a 20- to 22-gauge needle with a dose of 0.1 mL/kg and a subsequent 20-mL flush of 0.9% normal saline.

**Protocol for ^18^F-PSMA-1007 PET/CT**

Four megabecquerel/kg (1 becquerel = 2.7 × 10−11 curie [Ci]), with a minimum of 2 megabecquerel/kg and maximum of 400 megabecquerel, of ^18^F-PSMA-1007 were injected intravenously with the patients reclined in a standard PET uptake room. After a 2-hour uptake (range, 100 to 140 minutes from injection), patients were transferred to the PET scanner table. Patients were asked to void completely immediately prior to the scan. A standard PET acquisition was performed, beginning at the vertex and progressing downward to the toes. This was completed at 4 minutes per bed position, or equivalent bed movement speed depending on the scanner used and body part imaged. Over the legs, 2 minutes per bed position was conducted to minimize the time between PET and the subsequently acquired CT. The multiphase diagnostic CT scan of the same anatomic region was acquired for attenuation correction and diagnostic interpretation, with the administration of intravenous contrast, unless contraindicated. The serum creatinine (within 30 days of the study) and calculated eGFR were reviewed. If unavailable, serum creatinine was ascertained with an i-Stat 1 analyzer (Abbott Point of Care Inc) immediately before the scan. Patients were screened with a verbal questionnaire regarding history of intravenous iodinated contrast allergy. Intravenous contrast was administered for patients with eGFR of 40 mL/min/1.73 m2 or more and no history of anaphylactic allergic reaction to contrast. Iohexol (Omnipaque 350 [GE HealthCare]), 100 mL, was injected intravenously via a 20- to 22-gauge needle at a rate of 3.5 mL/s followed by 25 mL of normal saline at a rate of 3 mL/s. Helical CT images were obtained from the vertex to the toes at 25 seconds after injection and from the top of the diaphragm to the symphysis pubis at 80 seconds after injection. CT acquisition parameters for all CT scans were as follows: 120 kilovolts, 250 to 300 milliampere-seconds, and 50- to 70-cm field of view. Pitch, rotation speed, and slice thickness may vary slightly by scanner and body part imaged. The reconstructed PET images and correlative CT images were reviewed using Segami Oasis platform workstations (Segami Corporation).
