# Supplementary material for: Three-dimensional spatial localization and volume estimation of prostate tumors using 18F-PSMA-1007 PET/CT versus multiparametric MRI
Source: Eur J Nucl Med Mol Imaging. 2024 Dec 27;52(5):1642–8. doi: 10.1007/s00259-024-07021-0 (PMC11928431; doi:10.1007/s00259-024-07021-0)
Supplement: Supplementary file 3 — Supplementary file3 (DOCX 19 KB) [file 259_2024_7021_MOESM3_ESM.docx]

**Supplementary Table 1: Univariable analysis for correct tumor localization by ^18^F-PSMA-1007 PET/CT and MRI.**

|  | **^18^F-PSMA-1007 PET/CT** | |  | **MRI** | |
| --- | --- | --- | --- | --- | --- |
|  | **OR** | **p-value** |  | **OR** | **p-value** |
| **Age** | 0.99 | 0.54 |  | 0.99 | 0.70 |
| **Preop PSA Level** | 1.06 | 0.05 |  | 1.00 | 0.86 |
| **Prostate Volume** | 1.00 | 0.55 |  | 0.99 | 0.43 |
| **Tumor Volume** | **2.50** | **<0.001** |  | **2.14** | **<0.001** |
| **T stage** |  | **0.01** |  |  | **<0.001** |
| **≥ pT3** | **1.86** |  |  | **2.30** |  |
| **pT2** | **1.00** |  |  | **1.00** |  |
| **Tumor Focality** |  | **0.001** |  |  | **<0.001** |
| **Multifocal** | **0.12** |  |  | **0.10** |  |
| **Unifocal** | **1.00** |  |  | **1.00** |  |
| **Tumor Laterality** |  | **0.04** |  |  | **<0.01** |
| **Bilateral** | **0.20** |  |  | **0.15** |  |
| **Unilateral** | **1.00** |  |  | **1.00** |  |
| **Gleason Grade Group** |  | **<0.001** |  |  | **<0.001** |
| **≥ 3** | **20.96** |  |  | **50.00** |  |
| **2** | **5.53** |  |  | **9.47** |  |
| **1** | **1.00** |  |  | **1.00** |  |

OR = odds ratio. Age, pre-operative PSA, prostate volume, and tumor volume were analyzed as continuous variables for each increasing increment (year, ng/ml, cc).
